# Analysis of the equine “cumulome” reveals major metabolic aberrations after maturation in vitro

**DOI:** 10.1186/s12864-019-5836-5

**Published:** 2019-07-17

**Authors:** Jasmin Walter, Fabian Huwiler, Claudia Fortes, Jonas Grossmann, Bernd Roschitzki, Junmin Hu, Hanspeter Naegeli, Endre Laczko, Ulrich Bleul

**Affiliations:** 1 Clinic of Reproductive Medicine, Vetsuisse Faculty, University of Zurich, 8057 Zurich, Switzerland; 2 Functional Genomics Center Zurich, University and ETH Zurich, 8057 Zurich, Switzerland; 3 Institute of Pharmacology and Toxicology, Vetsuisse Faculty, University of Zurich, 8057 Zurich, Switzerland

**Keywords:** Oocyte, Cumulus, IVM, Proteomics, Metabolomics, Complement, Coagulation, Glucose, Oxygen, Purine

## Abstract

**Background:**

Maturation of oocytes under in vitro conditions (IVM) results in impaired developmental competence compared to oocytes matured in vivo. As oocytes are closely coupled to their cumulus complex, elucidating aberrations in cumulus metabolism in vitro is important to bridge the gap towards more physiological maturation conditions. The aim of this study was to analyze the equine “cumulome” in a novel combination of proteomic (nano-HPLC MS/MS) and metabolomic (UPLC-nanoESI-MS) profiling of single cumulus complexes of metaphase II oocytes matured either in vivo (*n* = 8) or in vitro (*n* = 7).

**Results:**

A total of 1811 quantifiable proteins and 906 metabolic compounds were identified. The proteome contained 216 differentially expressed proteins (*p* ≤ 0.05; FC ≥ 2; 95 decreased and 121 increased in vitro), and the metabolome contained 108 metabolites with significantly different abundance (p ≤ 0.05; FC ≥ 2; 24 decreased and 84 increased in vitro). The in vitro “cumulome” was summarized in the following 10 metabolic groups (containing 78 proteins and 21 metabolites): (1) oxygen supply, (2) glucose metabolism, (3) fatty acid metabolism, (4) oxidative phosphorylation, (5) amino acid metabolism, (6) purine and pyrimidine metabolism, (7) steroid metabolism, (8) extracellular matrix, (9) complement cascade and (10) coagulation cascade. The KEGG pathway “complement and coagulation cascades” (ID4610; *n* = 21) was significantly overrepresented after in vitro maturation. The findings indicate that the in vitro condition especially affects central metabolism and extracellular matrix composition. Important candidates for the metabolic group oxygen supply were underrepresented after maturation in vitro. Additionally, a shift towards glycolysis was detected in glucose metabolism. Therefore, under in vitro conditions, cumulus cells seem to preferentially consume excess available glucose to meet their energy requirements. Proteins involved in biosynthetic processes for fatty acids, cholesterol, amino acids, and purines exhibited higher abundances after maturation in vitro.

**Conclusion:**

This study revealed the marked impact of maturation conditions on the “cumulome” of individual cumulus oocyte complexes. Under the studied in vitro milieu, cumulus cells seem to compensate for a lack of important substrates by shifting to aerobic glycolysis. These findings will help to adapt culture media towards more physiological conditions for oocyte maturation.

**Electronic supplementary material:**

The online version of this article (10.1186/s12864-019-5836-5) contains supplementary material, which is available to authorized users.

## Background

Maturation of oocytes is the first step for in vitro production (IVP) of embryos across species. Oocyte maturation can occur under in vivo conditions where mature oocytes are collected from the donor for fertilization or under in vitro conditions. Usually, maturation in vitro is accompanied by decreased developmental competence among oocytes [1, 2]. The standard in vitro fertilization (IVF) protocol in human reproduction includes the ovarian stimulation of women with exogenous gonadotropins to mature oocytes in vivo [3]. Even though maturation and fertilization rates after in vitro maturation (IVM) are promising, IVM correlates with decreased implantation rates, increased miscarriage rates and increased live birth rates [3–5]. Therefore, candidates for IVM in human reproduction are mostly restricted to women at risk for ovarian hyperstimulation syndrome (OHSS) [3, 6]. For the equine species, direct comparisons of embryonic development after in vivo and in vitro are available. In one study in vitro matured oocytes were transferred into the oviduct for in vivo fertilization and further development. The results showed a highly decreased developmental capacity of the in vitro matured oocytes (9%) compared with that of the in vivo matured oocytes (82%) [1]. Blastocyst rates after intracytoplasmic sperm injection (ICSI) of in vitro matured oocytes achieved only up to 35%, which was distinctly lower than their in vivo matured counterparts (up to 70%) [2]. A special mystery in equine-assisted reproduction is the complete failure of classical IVF [7]. At present, this failure is reflected by only two foals born after classical IVF that originated from oocytes matured in vivo [8, 9]. All other equine IVP foals have been generated by ICSI [10, 11]. Whether the cause of this issue is located on the male or female side currently remains unclear [7, 12].

Available clinical data reflect the gap between in vitro matured and in vivo matured oocytes with regard to the developmental competence of oocytes. Fundamental research to elucidate altered metabolism during IVM is necessary to bridge this gap. Currently, high-throughput “Omics” technologies provide the opportunity to obtain a more global view on complex biological processes in reproduction [13–16]. The cumulus complex (CC) makes intimate contact with its oocyte and is required to obtain the maturational competence of the oocyte [17–21]. After maturation and fertilization, the CC is not required for further development; thus, these cells can serve as a unique source to noninvasively investigate metabolism during oocyte maturation [22]. Most of the available “Omics” studies on cumulus cells are transcriptomic analyses of pooled cumulus or cumulus oocyte complexes (COCs) that relate the gene expression profile to the developmental competence of the oocyte [23–30]. Other studies examined changes in the cumulus transcriptome between in vitro and in vivo matured COCs [31–34]. All these studies were performed in species other than the horse. An equine study on granulosa cells, which have a developmental origin similar to that of cumulus cells, observed age-related changes in their transcriptome [35]. Another transcriptomic study on equine granulosa and theca cells during dominant follicular development identified distinct expression profiles within these stages [36].

Studies focusing more closely on metabolism and the CC phenotype, e.g., using proteomics or metabolomics, are rare. One major limitation for the proteomics approach is the large amount of COCs required for analysis [37, 38] as enrichment of proteins prior to analysis is not possible. However, technical improvements for these techniques currently allow the analysis of small sample amounts [33, 39, 40]. Differences in the cumulus proteome through maternal ageing in humans [41], between cyclic and prepubertal whole porcine COCs [42, 43], and bovine cumulus cells and oocytes [44] were reported in studies using pooled CCs or COCs. However, beyond some practical benefits, pooling samples has some drawbacks such as masking of outliers, dilution of low abundance proteins and the loss of the possibility for the estimation of inter-individual variations within groups [45, 46]. These issues lead to the reduced applicability of pooled samples, especially for biomarker discovery [47]. Global protein expression profiling, without identification of altered protein spots, for human cumulus cells of single oocytes was performed in 2006 [48]. This previous study observed alterations in the protein expression profiles of cumulus cells under different stimulation protocols, as well as minor aberrations in fertilization outcomes using protein electrophoresis after metabolic labelling [48]. Only recently, intact-cellMALDI-TOF mass spectrometry (ICM-MS) in combination with top-down proteomics was investigated as tool for biomarker discovery in cumulus cells of single bovine oocytes [49]. Data on the equine cumulus cell proteome are not available in the literature. However, a characterization of the mare follicular fluid composition was performed during late follicular development using 2D-PAGE and mass spectrometry [50]. Similar methodology was used to characterize seasonal variation in equine follicular fluid [51].

Data on the cumulus cell metabolome are even scarcer across species. Comparison of in vitro matured with in vivo matured COCs revealed altered cellular metabolism-related genes along with increased triglycerides in bovine cumulus cells matured in vitro [34]. Glycosidic residues showed significant quantitative and qualitative differences in equine and porcine COCs after in vitro and in vivo maturation [52]. In the horse, maternal obesity caused alterations in the lipid fingerprint of preovulatory follicles and oocytes [53]. Individual analysis of lipid metabolism by desorption electrospray ionization mass spectrometry (DESI-MS) revealed distinct lipid profiles for individual oocytes and embryos [54–56].

Simultaneous profiling of the cumulus cell proteome and metabolome on the single COC level is a technical challenge but provides the unique opportunity to reveal the metabolism as close as possible to the phenotype. This multi-omics (“cumulomics”) approach was chosen for the characterization of aberrations in equine CC metabolism during maturation in vitro. A highly sensitive method allowed the analysis on the single COC level, which provides the unique opportunity in the future to directly correlate the “cumulome” with the developmental competence of the corresponding oocyte. The goal is to bridge the gap between in vitro and in vivo maturation and improve the culture conditions for IVM.

Equine oocytes also serve as an ideal model for translational research towards clinical human-assisted reproductive technologies. Both species usually develop a single dominant follicle of large volume and have a similar follicular phase and interovulatory interval [37, 57, 58]. Additionally, the timing of ovulation seems to be similar, occurring 36–37 h after human chorionic gonadotrophin (hCG) administration [57]. Another benefit of equine CCs for the study of metabolism during maturation for translational research is the large amount of cumulus compared to that in other species. This abundance of cumulus allows for the collection of enough material from single CCs for analysis. Therefore, the results of this study may contribute to improving human IVM conditions, which could save a wide range of women from the exhausting process of ovarian stimulation in the future.

## Results

### Proteome

**Fig. 1:**
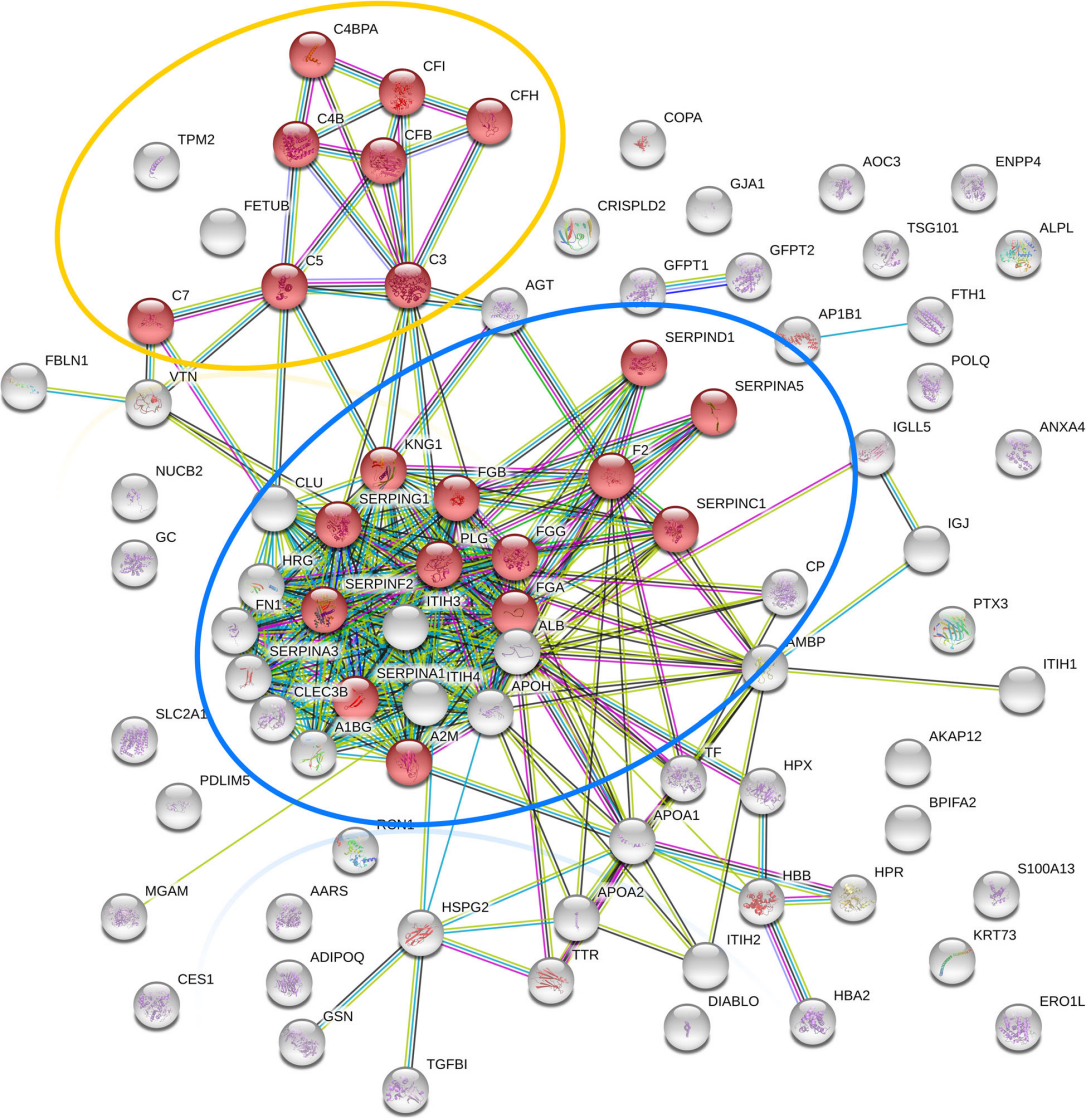
Interaction network of proteins underexpressed in in vitro matured cumulus complexes (interaction confidence: high (> 0.7), database matches *n* = 82). Highly enriched KEGG pathway in the group of underrepresented proteins is the complement and coagulation cascade (red nodes, pathway ID 04610; *n* = 21; false discovery rate 1.3e^− 32^). The proteins of the complement cascade are represented by the red nodes within the yellow circle (*n* = 8), whereas the proteins of the coagulation cascade are within the blue circle (*n* = 13)

**Fig. 2:**
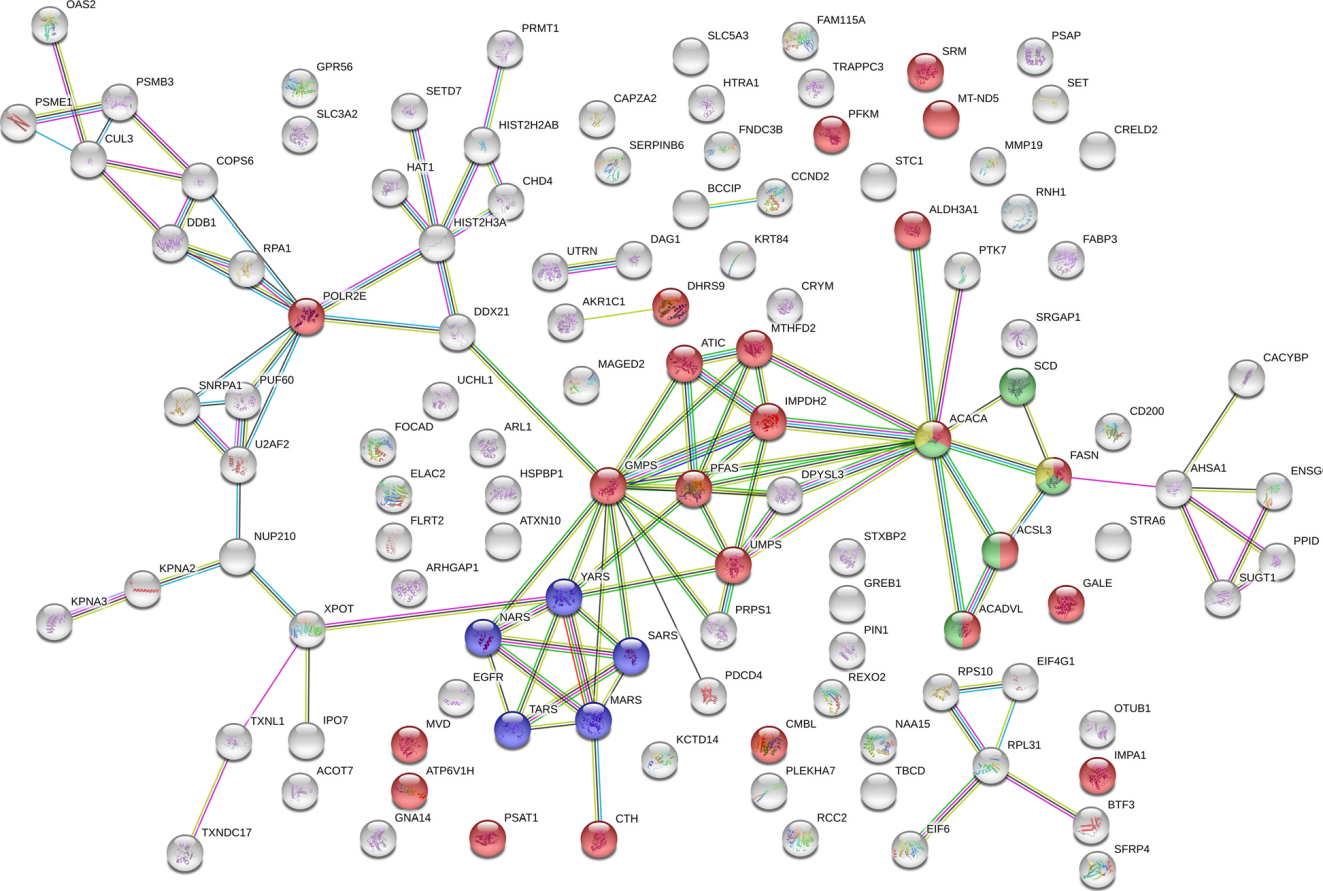
Interaction network of proteins overexpressed in in vitro matured cumulus complexes (interaction confidence: high (> 0.7), database matches *n* = 116). Enriched KEGG pathways in this group of proteins are metabolic pathways (red nodes, pathway ID 01100; *n* = 23; false discovery rate 3.6e^− 05^), aminoacyl-tRNA biosynthesis (blue nodes, pathway ID 00970; *n* = 5; false discovery rate 0.0006), fatty acid metabolism (green nodes, pathway ID 01212; *n* = 5; false discovery rate 0.0007) and fatty acid biosynthesis (yellow nodes, pathway ID 00061, *n* = 2, false discovery rate 0.04)

A total of 1811 quantifiable equine proteins (NCBI-Accessions) were identified in the 15 cumulus samples. For downstream analysis, the equine NCBI entries were blasted to human orthologous UniProt Accession, which yielded a total of 1714 unique entries. The proteome contained 216 differentially expressed proteins (*p* ≤ 0.05; FC ≥ 2; Table 1; Additional file 1: Table S1). Of these proteins, 95 were significantly underexpressed in vitro, and 86 of these proteins were linked to a unique orthologous human UniProt identitiy (ID; Fig. 1). In the in vitro group, 121 proteins (118 with unique orthologous human UniProt IDs) were significantly overexpressed (Fig. 2). Enrichment analysis of overrepresented Kyoto Encyclopedia of Genes and Genomes (KEGG) pathways was performed in the STRING-DB version 10.5 [59]. The pathway “complement and coagulation cascades” (KEGG ID 4610) was significantly overrepresented in the underexpressed proteins in in vitro matured cumulus (Fig. 1). Overrepresented KEGG pathways for the proteins overexpressed in in vitro matured cumulus were metabolic pathways (KEGG ID 01100), aminoacyl-tRNA biosynthesis (KEGG ID 00970), fatty acid metabolism (KEGG ID 01212) and fatty acid biosynthesis (KEGG ID 00061) (Fig. 2).

**Table 1:** Summary of the proteomics and metabolomics results. The counts of proteins and metabolomic compounds that were quantifiable and the counts that showed different abundances (p < 0.05; fold change (FC) > 2) in the in vitro matured cumulus samples compared to those in the in vivo matured cumulus samples are presented

Method	Quantifiable	Different abundance (p < 0.05; FC > 2)	Down in vitro	Up in vitro
Proteomics (proteins)	1811 (1714)^a^	216 (204)^a^	95 (86)^a^	121 (118)^a^
Metabolomics (compounds)	905	108 (28)^b^	24 (6)^b^	84 (22)^b^

^a^with unique orthologous human UniProt ID

^b^with putative metabolite ID

### Metabolome

The quantifiable metabolome contained 906 metabolic compounds; of these compounds, 108 showed a significant difference (*p* < 0.05; FC > 2) in abundance between the two maturation groups. Compared to the in vivo matured cumulus, the in vitro matured cumulus exhibited 84 compounds with a higher abundance and 24 compounds with a lower abundance (Table 1; Additional file 2: Table S2). Putative metabolite IDs were found for 6 compounds with lower abundance and for 22 compounds with higher abundance after IVM.

### Essence of the altered “cumulome” in vitro

**Fig. 3:**
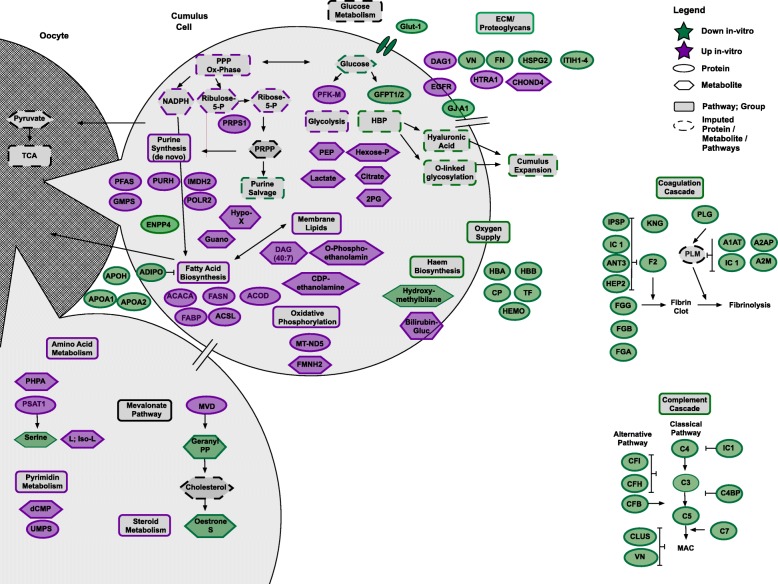
The essence of the altered “cumulome” after maturation in vitro. Schematic view on aberrant metabolism in cumulus cells after maturation in vitro compared to maturation in vivo. Mapped are 91 compounds with significantly different abundance in in vitro matured cumulus (78 proteins and 21 metabolites, full names are listed in Table 2). Relevant related proteins, metabolites or pathways not detected in this study were imputed (dashed lines). Items with higher abundance after maturation in vitro are coloured in purple, items with lower abundance in green. Circles surround proteins and hexagons metabolites

For an integrative view on the proteomic and metabolomic results, compounds with significantly different abundances were summarized in the following 10 metabolic groups (Table 2, Fig. 3): oxygen supply (down in vitro: 5 proteins, 1 metabolite; up in vitro: 1 metabolite), glucose metabolism (down in vitro: 3 proteins; up in vitro: 1 protein, 5 metabolites), fatty acid metabolism (down in vitro: 4 proteins; up in vitro: 5 proteins, 3 metabolites), oxidative phosphorylation (up in vitro: 1 protein, 1 metabolite), amino acid metabolism (down in vitro: 1 metabolite; up in vitro: 1 proteins; 2 metabolites), purine and pyrimidine metabolism (down in vitro: 1 protein; up in vitro: 7 proteins, 3 metabolites), steroid metabolism (down in vitro: 2 metabolites; up in vitro: 1 protein), extracellular matrix (down in vitro: 8 proteins; up in vitro: 3 proteins, 1 metabolite), complement cascade (down in vitro: 14 proteins) and coagulation cascade (down in vitro: 17 proteins). These 91 manually selected and grouped compounds of the overall 232 metabolites and proteins with unique IDs and significant differences between the two maturation groups are listed in detail in Table 2 and Fig. 3. All significantly different proteins (Additional file 1: Table S1) and metabolic compounds (Additional file 2: Table S2) are listed in the article supplements.

**Table 2:** The essence of the altered “cumulome” after maturation in vitro

Metabolic Groups with their Proteins/Metabolites	Abbreviation Figure	Proteomics: Orthologous Human Accession Uniprot	Proteomics: NCBI Accession	Anova (Progenesis)	Max fold change (Progenesis)	Up/Down in-vitro
		Metabolomics: HMDB ID	Metabolomics: Compound (m/z)			
1 Oxygen Supply (proteins: *n* = 5; metabolites *n* = 2)						
Hemoglobin A	HBA	HBA_HUMAN	NP_001078901.1	0.015	25.58	Down
Hemoglobin B	HBB	HBB_HUMAN	NP_001157490.1;XP_001504241.1	0.007	52.65	Down
Ceruloplasmin	CP	CERU_HUMAN	XP_001491539.1;XP_014587394.1;XP_014587022.1	< 0.001	140.94	Down
Transferrin	TF	TRFE_HUMAN	NP_001075415.2	0.003	10.25	Down
Hemopexin	HEMO	HEMO_HUMAN	XP_005612174.1	0.010	10.14	Down
Hydroxymethylbilane	Hydroxymethylbilane	HMDB01137	4.89_426.1165 m/z	0.001	16.86	Down
Bilirubin-Glucuronide	Bilirubin-Gluc	HMDB10332	6.37_741.2851 m/z	0.040	2.21	Up
2 Glucose Metabolism (proteins *n* = 4; metabolites n = 5)						
Glutamine-fructose-6-phosphate aminotransferase 1	GFPT1	GFPT1_HUMAN	XP_005599972.1	< 0.001	101.18	Down
Glutamine-fructose-6-phosphate aminotransferase 2	GFPT2	GFPT2_HUMAN	XP_014585826.1	0.024	2.80	Down
ATP dependend 6-phosphofructokinase muscle type	PFK-M	PFKAM_HUMAN	XP_005611133.1;XP_014596234.1	0.006	4.67	Up
Solute Carrier Family 2, facilitated glucose transporter member 1	GLUT1	GTR1_HUMAN	NP_001157443.1;XP_005607060.1	0.040	2.01	Down
Phosphoenolpyruvate	PEP	HMDB00263	6.23_166.9749 m/z	0.018	157.36	Up
D-Lactate / L-Lactate	Lactate	HMDB00190	6.41_111.0085 m/z	0.019	2.78	Up
Hexose-Monophosphate	Hexose-P	not specified	6.41_259.0217 m/z	0.039	6.10	Up
Citrate/Isocitrate	Citrate	HMDB00094	6.84_191.0191 m/z	0.024	4.04	Up
2-phospho-d-glyceric acid	2PG	HMDB03391	6.39_185.9927n	0.026	10.30	Up
3 Fatty Acid Metabolism (proteins *n* = 9; metabolites *n* = 3)						
Apolipoprotein A1	APOA1	APOA1_HUMAN	XP_005611649.1	0.001	30.68	Down
Apolipoprotein A2	APOA2	APOA2_HUMAN	XP_001503846.1	0.003	48.44	Down
Apolipoprotein H	APOH	APOH_HUMAN	XP_001499728.1	< 0.001	30.74	Down
Adiponectin	ADIPO	ADIPO_HUMAN	XP_001499564.1	0.009	8.64	Down
Acetyl-CoA carboxylase 1	ACACA	ACACA_HUMAN	XP_005597614.1;XP_001496980.1	< 0.001	6.07	Up
Fatty acid synthase	FASN	FAS_HUMAN	XP_014591306.1	0.001	2.64	Up
Long-chain-fatty-acid-CoA ligase 3	ACSL	ACSL3_HUMAN	XP_001915556.1	0.013	4.14	Up
Fatty acid binding protein	FABP	FABPH_HUMAN	NP_001157357.1;XP_005607059.1	0.001	46.96	Up
Acyl-CoA desaturase	ACOD	ACOD_HUMAN	XP_001500414.1	< 0.001	7.97	Up
O-Phosphoethanolamine	O-Phosphoethanolamine	HMDB00224	4.64_175.9943 m/z	0.002	2.76	Up
Diacyglycerol (40:7)	DAG (40:7)	not specified	3.61_647.5261 m/z	0.024	11.18	Up
CDP-Ethanolamine	CDP-Ethanolamine	HMDB01564	4.85_222.0249 m/z	0.021	2.75	Up
4 Oxidative Phosporylation (proteins *n* = 1; metabolites n = 1)						
NADH-ubiquinone oxidoreductase chain 5	Mt-ND5	NU5M_HUMAN	NP_007170.1	0.046	3.61	Up
reduced flavin mononucleotide	FMNH2	HMDB01142	7.19_439.0852 m/z	< 0.001	4.37	Up
5 Amino Acid Metabolism (proteins *n* = 7; metabolites n = 4)						
phosphoserine aminotransferase	PSAT1	SERC_HUMAN	XP_001496412.1	0.022	9.40	Up
L-Serine; D-Serine	SERINE	HMDB00187; HMDB03406	4.98_104.0352 m/z	0.008	3.22	Down
Phosphohydroxypyruvic acid	PHPA	HMDB01024	6.82_164.9577 m/z	0.000	19.00	Up
Leucine/Isoleucine	L; Iso-L	HMDB00687; HMDB00172	6.55_168.0429 m/z	0.003	7.84	Up
L-Cystine	Cystine	HMDB00192	4.81_221.0093 m/z	0.008	2.67	Up
Serine t-RNA ligase	SARS	SYSC_HUMAN	XP_005610429.1	0.007	2.17	Up
Tyrosine t-RNA ligase	YARS	SYYC_HUMAN	XP_014592779.1	0.015	2.06	Up
Asparaginyl t-RNA ligase	NARS	SYNC_HUMAN	XP_001488269.1	0.016	3.19	Up
Threonine t-RNA ligase	TARS	SYTC_HUMAN	XP_001498225.2;XP_001491149.3	0.005	2.11	Up
Methionine t-RNA ligase	MARS	SYMC_HUMAN	XP_001488941.1;XP_005611399.1	0.039	2.12	Up
Alanine t-RNA ligase	AARS	SYAC_HUMAN	XP_001501062.3	0.013	34.61	Down
6 Purin & Pyrimidin Metabolism (proteins *n* = 8; metabolites n = 3)						
Ribose-phosphate pyrophosphokinase	PRPS1	PRPS1_HUMAN	XP_001491425.1;XP_001489096.2;XP_001496502.1	0.011	2.09	Up
Phosphoribosylformylglycinamidine synthase	PFAS	PUR4_HUMAN	XP_001918417.1	0.006	14.09	Up
Bifunctional purine biosynthesis protein	PURH	PUR_9_HUMAN	XP_005610644.2	0.007	2.01	Up
Inosine-5′-monophosphate dehydrogenase 2	IMDH2	IMDH2_HUMAN	XP_001494600.4;XP_005600741.2	0.004	3.17	Up
Guanine Monophosphate Synthase	GMPS	GUAA_HUMAN	XP_014587031.1	0.003	2.23	Up
DNA-directed RNA polymerases I, II, and III subunit RPABC1	POLR2	RPAB1_HUMAN	XP_001496313.1;XP_014596768.1	0.026	3.93	Up
Bis(5′-adenosyl)-triphosphatase	ENPP4	ENPP4_HUMAN	XP_001502639.1	0.005	5.49	Down
Hypoxanthine	HypoX	HMDB00157	4.75_172.9976 m/z	0.014	2.62	Up
Guanosine	Guano	HMDB00133	4.63_318.0790 m/z	0.033	4.29	Up
Deoxycytidine monophosphate	dCMP	HMDB01202	6.89_343.9941 m/z	0.025	2.25	Up
Uridine 5′-monophosphate synthase	UMPS	UMPS_HUMAN	XP_001500089.4	0.009	3.17	Up
7 Steroid Metabolism (proteins n = 2; metabolites n = 2)						
Diphosphomevalonate decarboxylase	MVD	MVD1_HUMAN	XP_001488083.1;XP_014593934.1	0.007	6.21	Up
Growth Regulation by Estrogen in Breast Cancer Gene 1	GREB	GREB1_HUMAN	XP_014586792.1	0.002	4.26	Up
Geranyl Pyrophosphate	Geranyl-PP	HMDB01285	4.89_314.0589n	0.002	3.04	Down
Estrone Sulfate	Estrone-S	HMDB01425	6.98_385.0719 m/z	< 0.001	2.94	Down
8 Extracellular Matrix/Proteoglycans (proteins *n* = 11; metabolites n = 1)						
Vitronectin	VN	VTNC_HUMAN	XP_001504173.3	0.001	19.83	Down
Fibronectin	FN	FINC_HUMAN	XP_001489154.4	< 0.001	152.88	Down
Inter-alpha-trypsin inhibitor heavy chain H1	ITIH1	ITIH1_HUMAN	XP_001492576.1;XP_014587106.1	< 0.001	76.91	Down
Inter-alpha-trypsin inhibitor heavy chain H2	ITIH2	ITIH2_HUMAN	XP_001916967.1	< 0.001	45.40	Down
Inter-alpha-trypsin inhibitor heavy chain H3	ITIH3	ITIH3_HUMAN	XP_014587105.1	< 0.001	14.74	Down
Inter-alpha-trypsin inhibitor heavy chain H4	ITIH4	ITIH4_HUMAN	XP_005600608.1;XP_014587103.1	0.011	26.91	Down
Basement-membrane specific heparan sulfate proteoglycan core protein	HSPG2	PGBM_HUMAN	XP_014592730.1	0.014	3.17	Down
Epidermal Growth Factor Receptor	EGFR	EGFR_HUMAN	XP_005609210.2	0.009	2.24	Up
Dystroglycan	DAG1	DAG1_HUMAN	XP_001497663.1;XP_005600719.1;XP_005600720.1;XP_005600721.1	0.011	2.35	Up
Serine Protease HTRA1	HTRA1	HTRA1_HUMAN	XP_005602601.1	0.013	3.92	Up
Gap junction alpha 1 protein; Connexin-43	GJA1	CXA1_HUMAN	NP_001296155.1;XP_014584258.1;XP_014584259.1	0.008	2.69	Down
Chondroitin-4-sulphate	CHOND4	HMDB00652	4.75_496.0430 m/z	0.012	3.61	Up
9 Complement Cascade (proteins *n* = 14)						
Plasma protease C1 inhibitor	IC1	IC1_HUMAN; SERPING1	XP_001498388.1	< 0.001	56.92	Down
Complement 4 (A/B)	C4	CO4B_HUMAN	XP_001492943.1	0.007	7.78	Down
C4b-binding protein alpha chain	C4BP	C4BPA_HUMAN	XP_014594908.1	0.001	48.62	Down
C4b-binding protein alpha chain	C4BP	C4BPA_HUMAN	XP_001492582.2;XP_005609671.1;XP_005609672.1;XP_005609676.1;XP_005609677.1;XP_005609678.1;XP_014594955.1;XP_014594956.1;XP_014594957.1;XP_014594958.1	< 0.001	73.54	Down
C3 = Complement C3	C3	CO3_HUMAN	XP_014596564.1	0.005	9274.92	Down
C3 = Complement C3	C3	CO3_HUMAN	XP_001915589.1	0.001	748.41	Down
Complement C5	C5	CO5_HUMAN	XP_014591698.1;zz|FGCZCont0049_P01031|CO5_HUMAN	0.044	5.66	Down
Complement C7	C7	CO7_HUMAN	XP_005604353.1	0.004	33.92	Down
Complement factor I	CFI	CFAI_HUMAN	XP_014593434.1;XP_014593435.1;XP_014593436.1	< 0.001	16.51	Down
Complement factor B	CFB	CFAB_HUMAN	XP_001492602.1	0.008	33.85	Down
Complement factor H X1	CFH	CFAH_HUMAN	XP_001491754.3	0.019	70.12	Down
Complement factor H X5	CFH	CFAH_HUMAN	XP_005608148.2	0.002	12.74	Down
Clusterin	CLUS	CLUS_HUMAN	NP_001075413.1	0.049	3.44	Down
Vitronectin	VN	VTNC_HUMAN	XP_001504173.3	0.001	19.83	Down
10 Coagulation Cascade (proteins *n* = 17)						
Fibrinogen alpha chain	FGA	FIBA_HUMAN	XP_005607860.1	0.001	43.25	Down
Fibrinogen beta chain	FGB	FIBB_HUMAN	XP_003364583.1	< 0.001	55.46	Down
Fibrinogen gamma chain	FGG	FIBG_HUMAN	XP_001914833.2	< 0.001	36.00	Down
Kininogen-1	KNG	KNG1_HUMAN	XP_001499389.1;XP_005601930.1	0.008	14.66	Down
Prothrombin	F2	THRB_HUMAN	XP_001490892.3	0.001	6.66	Down
Heparin Cofactor 2	HEP2	HEP2_HUMAN; SERPIND1	XP_003365492.1	0.003	46.20	Down
Plasma serine protease inhibitor	IPSP	IPSP_HUMAN; SERPINA5	XP_001496026.2	0.031	44.21	Down
Antithrombin 3	ANT3	ANT3_HUMAN; SERPINC1	XP_014594947.1;zz|FGCZCont0237_P01008|ANT3_HUMAN	0.001	8.46	Down
Plasma protease C1 inhibitor	IC1	IC1_HUMAN; SERPING1	XP_001498388.1	<0.001	55.27	Down
Alpha-2-antiplasmin	A2AP	A2AP_HUMAN; SERPINF2	XP_001504386.3;XP_005597710.1	0.005	10.84	Down
Alpha-2-macroglobulin	A2M	A2MG_HUMAN	XP_014596179.1	0.015	49.65	Down
Alpha-2-macroglobulin	A2M	A2MG_HUMAN	XP_014596181.1;XP_014596182.1	0.014	40.92	Down
Alpha-2-macroglobulin	A2M	A2MG_HUMAN	XP_001499173.2	0.000	15.00	Down
Plasminogen	PLG	PLMN_HUMAN	XP_001500552.3	0.027	16.17	Down
Alpha-1-antiproteinase 2 precursor	A1AT	A1AT_HUMAN	NP_001108005.1	0.001	35.00	Down
Alpha-1-antiproteinase 2 precursor	A1AT	A1AT_HUMAN	XP_005605481.1	0.001	33.45	Down
Alpha-1-antiproteinase 2 precursor	A1AT	A1AT_HUMAN	XP_001495905.2	0.001	27.21	Down

## Discussion

From a technical point of view, in this “cumulomics” study, a simultaneous profiling of the cumulus cells proteome and metabolome from the very same samples was performed for the first time. This technical development opened the gate to unravel the metabolic phenotype of cumulus cells under different maturation conditions. The analysis was performed on the level of single COCs, which holds the unique potential to directly correlate the “cumulome” with the developmental competence of the corresponding oocyte. A novel combination linking methanol based metabolite extraction with filter-aided sample preparation (FASP) for proteomics was adapted to for the minute sample amount of single CCs [39]. Thus far, only studies focusing on single “Omics” technologies have been available. Recently, intact MALDI-TOF mass spectrometry was used to analyse the proteome of individual bovine oocytes, cumulus cells and granulosa cells; a total of 439 shared peaks were detected, and identification of the peaks was performed using top-down proteomics on protein extracts of pooled samples [49]. An analysis of the lipid profile of single bovine oocytes and embryos was previously conducted with DESI-MS [54–56]. For this study, a combination of bottom-up proteomics by nano-HPLC MS/MS and metabolome analysis by UPLC-nanoESI-MS in negative mode was used for the analysis of individual CCs. The detected “cumulome” included 1811 quantifiable proteins as well as 905 quantifiable metabolic compounds. Therefore, the analytical technique of the “cumulomics” approach proved to be a highly sensitive “holistic approach”, which can characterize metabolic alterations on the single COC level.

Twenty-eight metabolites with putative metabolite IDs and 204 proteins with unique UniProt IDs were significantly different between the two maturation groups (Table 1). These alterations affect a wide variety of metabolic pathways (Fig. 3). The experimental design used an available pool of slaughtered animals; thus, there was certain heterogeneity with regard to donor mares (e.g. breeds and ages) and follicles (Additional file 4). This reflects the typical situation when immature COCs are collected for assisted reproduction from client mares or from slaughterhouse animals for research purposes. Therefore, factors as age and follicular size cannot fully be ruled out as confounding factors. Mean mare age was higher in the in vitro group, still not significantly different. Donor age was already shown to influence the equine granulosa cells transcriptome [35] and human cumulus proteome [41]. Gene expression profiles in granulosa cells vary throughout folliculogenesis, for the bovine species this was illustrated on the GranulosaIMAGE interactive web interface [60]. For the in vitro matured group it is not possible to specify exactly the developmental phase of the donor follicles, which can also impact the results. The picture of altered metabolism after IVM is of course influenced by maturation conditions. Differences in media composition, gas concentrations or cell handling would obviously affect the altered “cumulome”. Some of these potential effects are discussed in relevant chapters in the discussion.

### Oxygen supply

An interesting group of proteins underexpressed in in vitro matured cumulus is related to oxygen supply; first and foremost among these proteins is hemoglobin (Hb) A and B. The expression of Hb in non-thyroid cells was illustrated through recent advances in Hb research [61]. Recent studies documented hemoglobin B (HBB) expression in murine and human cumulus cells and exhibited underrepresentation of HBB after IVM [49, 62–64]. The function of HBB in these cells remains unknown. HBB can ensure the necessary oxygen supply for the oocyte in the reduced oxygen environment of the maturing follicle [61, 65]. Under in vivo conditions, the estimated oxygen concentrations in the follicle were approximately 1–5% [66], which is tremendously lower than the common 20% under IVM conditions [67]. Hypoxia induces the expression of HBB through hypoxia inducible factor 1 (HIF-1) [61, 68]. Higher levels of hypoxia inducible factor 1 α (HIF1α) protein were detected in cumulus cells exposed to low oxygen compared to those exposed to 20% oxygen during IVM [64]. Therefore, the high oxygen concentration under IVM conditions seems to be responsible for the underrepresentation of hemoglobin A (HBA) and HBB under in vitro conditions in this study. Hemopexin, a scavenger enzyme for Hb, was also underrepresented in the in vitro matured cumulus. When heme is released through degradation of Hb, it is transferred to hemopexin. Therefore, hemopexin acts as a detoxifying agent for free heme with its pro-inflammatory and pro-oxidant effects.

Another hint for the potential oxygen binding/transporting role of Hb in the *cumulus oophorus* is an analyte that was also significantly underrepresented in the in vitro matured group and seems to match hydroxymethylbilane (HMB), a precursor of heme biosynthesis. The transcripts of enzymes involved in heme biosynthesis were previously detected in COCs including the enzyme directly related to HMB synthesis (Hmbs; hydroxymethylbilane synthase) [63].

The in vitro underrepresented proteins ceruloplasmin and transferrin work together in the transport of Fe^2+^, which is required for heme synthesis. The underrepresentation of these proteins contribute to the observed underrepresentation of HBA, HBB and HMB. The simultaneous underrepresentation of proteins and metabolites involved in in vitro matured COCs strengthens the hypothesis regarding an oxygen binding role of hemoglobin within the COC, which seems to be deficient in in vitro matured cumulus cells. Nevertheless, heme is a constituent of hemoglobin and is required for cytochromes, cholesterol biosynthesis (see below) and hydrogen peroxide degradation (heme containing monofunctional catalases). Therefore, other functions of this molecule in this setting are also possible.

Heme catabolism results in the production of bilirubin, a yellow compound that seems to be responsible for the yellow colour of follicular fluid in humans [69]. One analyte overrepresented in in vitro matured cumulus (6.37_741.2851 m/z) can be attributed to the conjugated form of bilirubin, bilirubin glucuronide. The higher abundance can be a result of increased heme catabolism, therefore contributing to the underrepresentation of Hb in IVM cumulus in this study.

In summary, the underrepresentation of all these proteins and metabolites related to oxygen supply in in vitro matured cumulus cells play an important role in the further developmental potential of COCs. This underrepresentation may be one reason for the impaired developmental competence of in vitro matured COCs. The addition of ferrohemoglobin (FE^2+)^ to the IVM medium improved blastocyst rates [63]. Moreover, the in vitro condition used for the present study included a high oxygen environment (20% oxygen), which seems to result in the underrepresentation of these proteins compared to that in COCs matured in vivo under low oxygen conditions.

### Glucose metabolism

Glucose metabolism plays a central role in the altered compounds under in vitro conditions. First, glucose transporter member 1 (GLUT1) was significantly underrepresented in in vitro matured cumulus. GLUT1 was the highest expressed glucose transporter at the mRNA and protein levels in mouse cumulus cells where insulin stimulation resulted in glucose uptake [70]. Bovine cumulus responded to hypoxia during IVM through upregulation of GLUT1 gene expression (Slc2a1), and HIF1α seems to play a role in mediating this response [64]. For equine cumulus cells, the expression of the GLUT1 gene (Slc2a1) was previously documented, with significantly higher abundance in expanded CC than in compact cumulus cells [71]. These studies corroborate the observed GLUT1 underrepresentation after maturation under the hyperoxic (20%) in vitro condition in this study.

The importance of glucose metabolism for the maturation of COCs is well described in the literature. Oocytes possess only a limited capacity to metabolise glucose; they depend on cumulus cells for the valorisation of glucose [72]. The glycolytic compounds upregulated in in vitro matured cumulus cells are phosphoenolpyruvate (PEP) and lactate (Table 2, Fig. 3). The higher abundance of these potential metabolites indicates an increased glycolytic rate compared to that in in vivo conditions.

This theory is supported by overexpression of ATP dependent 6-phosphofructokinase muscle type (PFK-M), the main rate-limiting enzyme in glycolysis, after maturation in vitro. PFK-M is regulated by ATP concentrations; a high ATP/ADP ratio downregulates the glycolytic rate. Therefore, the overrepresentation after IVM seems to be the result of an energy (ATP) deficit or increased ATP consumption. For the equine species, reduced glucose metabolism was detected for expanded COCs. These COCs also exhibited higher maturational competence (50% versus 21.7%). These expanded COCs showed significantly lower glucose consumption along with decreased pyruvate and lactate production [71].

Alternatively, the rate-limiting enzyme of the hexosamine biosynthetic pathway (HBP) is glutamine-fructose-6-phosphate aminotransferase 1 and 2 (GFPT1 and 2), which was significantly underexpressed in in vitro matured cumulus cells. HBP participates in the production of hyaluronic acid and products for O-linked glycosylation of proteins [72, 73]. The role of HBP can explain the general observation of better expanded CCs after maturation in vivo compared to that in vitro. For the interpretation of these alterations in glucose metabolism, the available glucose under the two experimental conditions must be taken in account. The concentration of glucose in the natural surroundings of COCs, the follicular fluid, is usually in the range of plasma levels. Compared to other species, equine follicular fluid contains slightly higher glucose concentrations, ranging from 4.7 mmol/l in small follicles, with a drop to 3.3 mmol/l in larger follicles [74]. DMEM/F12, used for IVM in this study, contains an almost 4-fold higher (17.5 mmol/l) glucose concentration compared to that in follicular fluid. The switch from TCM199-based maturation medium (with a more physiological glucose concentration) to DMEM/F12-based maturation medium for equine oocytes resulted in significantly better cleavage and blastocyst rates, without alteration of the maturation rates [75]. Therefore, the high glucose concentration during IVM seems to be beneficial for the developmental competence of equine oocytes. Nevertheless, a substantial discrepancy for the “cumulome” between in vivo and in vitro matured COCs was observed. In summary, energy generation in vivo does not seem to heavily depend on glycolysis unlike that in vitro.

### Fatty acid metabolism

Under the experimental condition of this study, fatty acid synthesis seems to be upregulated after maturation in vitro*.* Three main enzymes involved in fatty acid synthesis (ACSL; ACACA; FASN) were overrepresented in in vitro matured cumulus. This overrepresentation is most likely the result of an insufficient supply of fatty acid in the maturation medium compared to that in the follicular environment in vivo. The only source of fatty acids in the medium was the supplemented serum, which contains less essential linoleic acid than is contained in the DMEM/F12-based medium used. In potential support of this hypothesis, three components of lipoproteins (APOA1 and 2; APOH) were underrepresented after IVM. These proteins are involved in lipid transport and storage and play an important role in providing lipids for the COCs. APOA1 was identified as crucial component of mare follicular fluid throughout the reproductive seasons [51]. Lipid metabolism is an important player in COC metabolism and oocyte developmental competence [76]. The special role of cumulus cells in supporting oocyte developmental competence through fatty acid synthesis and oxidation was described in a bovine study [77]. Meiotic resumption was compromised by inhibition of ß-oxidation in mouse [78, 79] and cow oocytes [77]. There was a close link to glucose consumption as inhibition of ß-oxidation resulted in increased glucose consumption. This finding are reflected in the altered equine “cumulome” of in vitro matured COCs where increased protein amounts for fatty acid synthesis and glycolysis were major findings in the altered metabolism after IVM. Interestingly, very similar results were observed in a bovine study where an increase in the lipid content of in vitro matured cumulus cells was accompanied by upregulation of genes related to glycolysis and fatty acid synthesis, whereas ß-oxidation was decreased after maturation in vitro [34]. Adiponectin (ADIPO) was also upregulated in in vitro matured cumulus cells. This regulator prevents energy deficits in cells, which was present in in vitro matured COCs as indicated by a lower ATP/ADP ratio compared to that in in vivo matured COCs [34].

In addition to the altered lipid related proteome, metabolic compounds that can be related to metabolites in fatty acid metabolism and membrane lipids were upregulated after maturation in vitro. One analyte can be attributed to DAG (40:7), a diglyceride that can be a precursor for triglycerides, products of membrane lipid degradation or serve as second messengers (https://pubchem.ncbi.nlm.nih.gov). Two other potential compounds upregulated in vitro, **O-** phosphoethanolamine and CDP-ethanolamine, belong to the glycerophospholipid metabolism pathway (KEGG pathway 00564). These metabolites are precursors for phospholipids or products of phospholipid breakdown (https://pubchem.ncbi.nlm.nih.gov). Metabolomic analysis of bovine IVM COCs and denuded oocytes after IVM revealed that cumulus cells modulate the lipid profile. Triacylglycerols and phospholipids were higher in COCs than in denuded oocytes [80]. Phospholipid metabolism compounds accumulated in bovine IVM medium during maturation, which makes production and secretion by COCs into the medium likely [81]. In summary, the presented equine results revealed major aberrations in fatty acid metabolism after maturation in vitro on both levels of the analysed “cumulome”. The synthesis of fatty acids in cumulus cells seems to compensate for the insufficient supply of fatty acids in COCs in vitro.

### Oxidative phosphorylation

Oxidative phosphorylation occurs at the mitochondrial membrane and produces energy (ATP) using the electrons generated from glucose in the TCA cycle. A central part in the mitochondrial respiratory chain is NADH-ubiquinone oxidoreductase chain 5 (Mt-ND5), which was overrepresented after maturation in vitro. This enzyme transfers electrons from NADH out of the TCA cycle to the respiratory chain. The intermediate electron acceptor for the enzyme is FMN, which is reduced to FMNH2 in the electron transport chain and was also overrepresented in in vitro matured cumulus cells. The overrepresentation of these enzymes in oxidative phosphorylation might be the consequence of an energy deficit in COCs during maturation in vitro.

### Amino acid metabolism

The altered metabolome after maturation in vitro also indicates potential aberrations in the amino acid distribution. Phosphohydroxypyruvate (PHPA) and phosphoserine aminotransferase (PSAT1), which catalyse the conversion to phosphoserine in the serine biosynthesis pathway, were overrepresented after maturation in vitro. This result can be explained by the underrepresentation of a compound attributed to serine after IVM. Serine is not an essential amino acid; thus, the potential lack of this compound is most likely the result of a higher demand e.g., for nucleotide synthesis (see the section Purine and Pyrimidine Metabolism). The overrepresentation of compounds in serine biosynthesis support this hypothesis for an increased serine demand in vitro. Opposed to serine, a metabolic compound that can be attributed to leucine/isoleucine was overrepresented after maturation in vitro. These aberrations in the equine “cumulome” after IVM raise the question of whether higher concentrations of serine in maturation media contribute to more physiological culture conditions for COC maturation.

### Purine and pyrimidine metabolism

In in vitro matured cumulus, metabolomic compounds (3) and proteins (7) that can be associated with purine and pyrimidine metabolism were significantly overrepresented. The first important protein linking the pentose phosphate pathway (PPP) with purine and pyrimidine biosynthesis is ribose-phosphate pyrophosphokinase (PRPS1), which was overrepresented after maturation in vitro. PRPS1 produces the metabolite phosphoribosyl pyrophosphate (PRPP) from the substrate ribose-5-phosphate from the PPP. PRPP is used for purine and pyrimidine biosynthesis as well as purine salvage pathways. Purine and pyrimidine metabolism in cumulus cells plays an important role in the orchestration of meiotic resumption. The increase in enzymes involved in purine biosynthesis in the cumulus of equine in vitro matured cumulus cells can be a result of decreased purine salvage compared to that in in vivo matured cumulus cells. Energy requirements (ATP) for the purine salvage pathways are distinctly lower (5x) as for de novo synthesis [82]. However, the rate of salvage pathway depends on PRPP concentration; therefore, it can be hypothesized that in vitro maturated cumulus cells suffer from a lack of PRPP. Upregulation of enzymes in de novo synthesis tries to compensate for this lack of PRPP. This issue would result in increased energy requirements for nucleotide production compared to those in in vivo conditions, which explains the shift in glucose metabolism towards the PPP (PRPP production) and glycolysis (ATP production).

### Steroid metabolism

Diphosphomevalonate decarboxylase (MVD), an enzyme catalysing the last reaction in the mevalonate pathway, was overrepresented in in vitro matured cumulus. The mevalonate pathway converts mevalonate in sterol isoprenoids (cholesterol) or non-sterol isoprenoids (e.g., heme-A or ubiquinone) [83]. MVD is responsible for the synthesis of isopentenyl diphosphate (IPP). IPP is condensed with dimethylallyl diphosphate to form geranyl diphosphate (geranyl-PP), a metabolite attributed to a metabolomic compound that was underrepresented in vitro [84]. MVD shows the highest expression in the liver for cholesterol production [84], but its expression was also documented in mouse ovarian follicles in the antral stage [85]. Cumulus cells provide cholesterol to their oocytes, which is transferred through gap junctions. Synthesis of cholesterol in cumulus cells is stimulated by oocyte secreted factors [86]. Cholesterol is the precursor for steroidogenesis in mammalian cumulus cells during maturation [87, 88].

Another metabolite MS signal underrepresented after maturation in vitro was assigned to the steroid hormone oestrone sulfate (oestrone-s). Estrone-s is biologically inactive, but through sulfotransferases, it can be converted into biologically active unconjugated estrone. Therefore, estrone-s can serve as reservoir for estrone. Expression of local steroid sulfatases was documented in human cumulus cells [89]. The results of this study revealed a deficit in potential metabolites in mevalonate/steroid metabolism with upregulation of a key enzyme of the mevalonate pathway in in vitro matured cumulus cells. This finding can be interpreted as the struggle of IVM COCs to produce sufficient steroids in compensation of the missing steroidogenic follicular surrounding.

Hemoglobin A (HBA) is a non-steroid isoprenoid product of the mevalonate pathway, which was also underrepresented after maturation in vitro (see the section oxygen supply). Steroids can stimulate porphyrin (heme) biosynthesis [90] and increase Fe incorporation [91]. Therefore, the lack of steroids in in vitro matured cumulus cells can also result in suppressed heme biosynthesis.

### Extracellular matrix/proteoglycans

Extracellular matrix (ECM) composition plays an important role in the fertilization of the oocyte. The ECM expands during the maturation process, and only successfully expanded ECM around cumulus allows correct adhesion and oviductal pick-up of the COC, influencing sperm motility and adhesion as well as fertilization [92–95]. Therefore, it is not surprising that a substantial portion of alterations in the “cumulome” after IVM is associated with ECM composition.

Through the gonadotrophin surge, cumulus cells are stimulated to form a hyaluronan-rich ECM responsible for the expansion of the CC [96]. However, inter-alpha-trypsin-inhibitor heavy chains 1–4 (ITIH1–4) were underrepresented in in vitro matured cumulus. This group of proteins is responsible for retaining hyaluronic acid (HA) in the cumulus matrix for expansion [97, 98]. A putative upregulated compound in in vitro matured CCs was chondroitin-4-sulfate (CHOND4). For mice, the binding of HA during maturation in the *cumulus oophorus* was hypothesized to be enabled by the release of chondroitin sulfate into the culture medium (in vitro) or follicular fluid (in vivo). This exchange of chondroitin sulfate with HA leads to stabilization of the cumulus ECM through a covalent interaction of HA and ITIH [99]. These results are in accordance with the presented equine “cumulome” data where ITIHs were underrepresented after IVM and CHOND4 was overrepresented. That is, during IVM, CHOND4 might be not sufficiently released into the culture medium and replaced by HA. Mouse in vivo matured COCs were more resistant to shear stress than in vitro matured COCs, which supports this hypothesis [99].

Another protein underrepresented after IVM in this context was heparan sulfate proteoglycan 2 (HSPG2, Perlecan). HSPG2 is a core protein attached to three glycosaminoglycan chains (heparan sulfate or chondroitin sulfate). HSPG2 is a main component of basement membranes [100], but its expression in cumulus cells of germinal vesicle stage oocytes from women [101] and granulosa cells from cows [60] was previously documented. Heparan sulfate proteoglycan expression peaked in rat preovulatory granulosa cells where the core proteins, such as HSPG2, remaining constant throughout the cycle. These proteoglycans can bind and activate antithrombin III (also underrepresented in in vitro matured cumulus); thus, they also possess a role in the control of proteolysis and fibrin formation [102] (see chapter coagulation cascade).

Vitronectin (VN), which was underrepresented after IVM, is a glycoprotein found in ECM that promotes cell adhesion. VN plays a role in the cytolytic complement pathway through the regulation of membrane attack complex (MAC) formation [103] (Fig. 3). VN in bovine cumulus ECM showed a negative effect on sperm motility [104] as well as a dose dependent effect on oocyte-sperm interactions [105]. Another important protein in ECM that was underrepresented after IVM is fibronectin (FN), another adhesive glycoprotein. FN is secreted by cumulus cells during maturation [93], and induced capacitation in human sperm [106]. FN content in human follicular fluid seemed to be a marker for oocyte quality, maturity and fertilization capability [107, 108]. Recently, for mice COCs, the FN-integrin pathway was shown to play an important role in cumulus expansion during ovulation [109]. In human cumulus samples, FN in younger women was higher than that in older women [110]. Both findings illustrate that FN expression in cumulus cells is positively associated with the oocyte developmental potential. A novel specific splice variant of bovine FN was observed in cumulus cells, which raised the hypothesis of a special function of this variant in cumulus cells [111]. In vitro matured equine oocytes also possess a reduced capacity for further development, and reduced expression of FN was observed in this study. These data contribute to the available literature and indicate a role of FN as a cumulus marker for the developmental competence of corresponding oocytes. Additionally, a role in the mediation of equine sperm-oocyte contact seems likely but needs further investigation.

Interestingly, serine protease HTRA1 was significantly overrepresented in the in vitro matured cumulus. This protease has a variety of targets but especially degrades FN in the ECM. ECM remodelling by HTRA1 affects a variety of pathobiological conditions such as osteoarthritis, cancer, and Alzheimer’s disease [112]. In human cumulus cells, HTRA1 expression was significantly higher than that in granulosa cells [113], which can be attributed to a special role of this protein in cumulus cell ECM production. The upregulation of this protein in in vitro matured cumulus might be responsible for the underrepresentation of FN after maturation in vitro.

Another protein that was underrepresented in in vitro matured cumulus and plays an important role in the ability of oocytes to achieve their full developmental competence is gap junction alpha 1 protein (GJA1; Connexin-43 (CX43)). Oocytes share close bidirectional communication with their surrounding oocytes. The exchange of small molecules and cellular coupling via gap junctions between cumulus cells and their surrounding oocyte is especially important [114]. The crucial role of CX43-mediated cumulus oocyte communication for the meiotic maturation of oocytes was documented for bovine [115] as well as human oocytes [116]. The presence of open gap junctions in granulosa cells is necessary for the maintenance of the oocyte in meiotic arrest. Granulosa cells deliver cGMP through gap junctions to the oocyte; this process maintains high levels of cAMP in the oocyte high as well as meiotic arrest [117]. Differences in gap-junctional coupling of equine oocytes were found with regard to breeding season; 90% coupling was detected in breeding season versus 55% interrupted communication in the non-breeding season [118]. Meiotic resumption of equine oocytes seemed to be associated with a decline in CX43 protein in cumulus cells, with significantly higher levels in in vitro than in in vivo matured cumulus cells [119]. Nonetheless, available equine gene expression data for GJA1 (CX43) in equine cumulus cells showed no variation between in vivo and in vitro maturated cumulus cells. Therefore, altered protein expression seems to be a result of post-transcriptional regulation [120]. For bovine oocytes, the expression level of CX43 in cumulus cells significantly correlated with the developmental competence of the corresponding oocyte [114]. Whether this correlation is also valid for the equine species needs further investigations.

Thus far, the discussed alterations with regard to ECM composition were mostly compounds identified as underrepresented after maturation in vitro. Some candidates showed an opposite trend with overrepresentation after IVM. One of these candidates, dystroglycan (DAG1), is an ECM glycoprotein that is poorly characterized in follicular cells compared to some of the previously discussed molecules. In porcine granulosa cells, DAG1 showed a decrease in concentration with increasing follicle size [121].

Overrepresented epidermal growth factor receptor (EGFR) is the transmitter of extracellular events in the cells. EGFR kinase activity is responsible for gap junction closure in response to LH, which results in meiotic resumption [117]. The functionality of EGFR seems to be one key factor for the oocyte to acquire developmental competence [122]. The addition of epidermal growth factor (EGF) to the maturation medium of equine oocytes significantly increases maturation rates [123]. For bovine oocytes, EGFR expression in cumulus cells seems to be a marker for oocyte developmental competence [23]. All these data indicate a positive effect of EGFR activity on oocyte developmental competence. The overexpression of EGFR receptor in the in vitro matured samples can be a result of the underrepresentation of a metabolic compound assigned to oestrone-s (underrepresented in in vitro matured cumulus, see the chapter steroid metabolism). In breast cancer, oestrogen was responsible for maintaining low levels of EGFR expression [124]. Another explanation for this upregulation delivers the addition of 50 mg/ml EGF to the maturation medium. EGF showed a positive effect on maturation in many species and is routinely added to equine maturation media [125]. In hepatic epithelial cells, EGF increased EGFR mRNA 3–5-fold [126].

### Complement Cascade

The most dominant proteome result is the massive overrepresentation of the complement and coagulation cascades (pathway ID 04610; String Enrichment Analysis *n* = 21 proteins; complement cascade *n* = 8 proteins; false discovery rate 1.3e^− 32^) in the group of proteins downregulated in the in vitro matured cumulus. In total, 11 unique proteins are associated with the complement cascade (Table 1, Fig. 3). The complement system is part of the innate immune system and includes 30 proteins overall. In this study, proteins of the classical and alternative pathway of complement activation are represented in the group of proteins with significantly lower expression. In addition to the complement factors C3, C4, C5 and C7 and the positive regulator CFB, negative regulators of the complement cascade (IC1, C4BP, CFI, CFH, CLUS, VN) belonged to the underexpressed proteins in the in vitro matured cumulus cells (Fig. 3).

An active complement system in the follicular fluid is relevant for ovulation in vivo [127–129]. Previous studies on the follicular fluid of mares revealed the presence of complement proteins in follicular fluid [50] with seasonal variation [51]. Several complement proteins seem to be shed within cell secreted vesicles into the follicular fluid of mares [130]. Confirmation of gene expression for several complement proteins in human granulosa cells supports the hypothesis that these cells are able to actively secrete complement factors [131]. For component C3, available studies indicate a role in the developmental competence of the oocyte [132–134]. The reduced presence of 11 proteins of the complement cascade supports the hypothesis that the lack of complement proteins is responsible for the reduced developmental competence of equine oocytes matured in vitro. Moreover, complement component 3 plays a role in sperm-oocyte-interactions [135, 136]. Transferring this context to the equine species, the lack of complement factors in equine CCs after IVM contribute to the inability of equine sperm to fertilize COCs. Further experiments are necessary to clarify the role of the complement system in the maturation process and the mediation of equine sperm-oocyte interactions.

### Coagulation Cascade

In in vitro matured cumulus, a wide variety of factors participating in the coagulation cascade were significantly underrepresented (*n* = 13; Table 2; Fig. 3). Three fibrinogen chains (A,B,G), which are converted into fibrin in the coagulation process by thrombin (F2a), belong to this protein group. Thrombin is derived through enzymatic cleavage of prothrombin (F2), which was also underrepresented in in vitro matured cumulus. Additionally, the following group of thrombin inhibitors with anticoagulant activity was underrepresented: heparin cofactor 2 (HEP2), plasma serine protease inhibitor (IPSP), antithrombin 3 (ANT3), and plasma protease C1 inhibitor (IC1). Moreover, proteins involved in fibrinolysis were underrepresented. Plasmin (PLM) is the enzyme responsible for fibrinolysis; the precursor of PLM, plasminogen (PLG), as well as the following proteins with inhibitory effects on PLM were underrepresented in the in vitro matured group: alpha-2-antiplasmin (A2AP); alpha-2-macroglobulin (A2M) and alpha-1-antiproteinase 2 precursor (A1AT). In summary, the in vitro matured cumulus appears to lack fibrinogen as well as the two main progenitors for coagulation (F2) and fibrinolysis (PLG). The shortage of these factors seems to be the result of massive consumption during COC collection or IVM. In support of this hypothesis, inhibitory factors for coagulation (IPSP, IC1, ANT3, and HEP2) as well as inhibitory factors for fibrinolysis (A1AT, A2AP, IC1, and A2M) were also underrepresented in the in vitro matured cumulus.

The coagulation system is a dominant part of equine follicular fluid [50, 51]. F2 and PLG were detected mainly during spring anovulatory season. Fluctuation in follicular coagulation factors throughout the reproductive season delivers explanations for different incidences for hemorrhagic anovulatory follicles [51]. Fibrinogen concentrations in equine follicular fluid were measured 40% higher than those in plasma [137]. Therefore, there must be local production/secretion of fibrinogen from follicular cells. Analysis of gene expression from human granulosa and cumulus cells of preovulatory follicles revealed the selective expression of fibrinogen in granulosa cells [138]. Coagulation system proteins that were upregulated in follicular fluid from women who underwent IVF successfully included fibrinogen, kininogen-1, prothrombin and coagulation factor XII [139]. Regarding the role of the coagulation system on oocyte maturation, available data are rare. The analysis of bovine cumulus cell gene expression after IVM revealed a significant enrichment of genes (*n* = 5) involved in the complement and coagulation pathway [32]. Through upregulation of the transcriptional machinery for these proteins, the cells try to compensate for the increased consumption of these proteins during IVM. Beyond the more general role of the coagulation system, maturation-specific functions in the fertilization process are possible. Antithrombin III (Serpin C1) plays a role as chemoattractant for sperm [140]. A derivate of A2M stimulates spermatozoa-zona pellucida binding in the human cumulus matrix [141]. Therefore, the study revealed specific candidates that hold the potential to improve the fertilization of equine COCs.

## Conclusions

In summary, according to these key findings, metabolism under in vitro conditions seems to focus on fuelling cells with energy via aerobic glycolysis as important candidates involved in oxygen supply and glucose metabolism were altered. This alteration can be the result of the culture system used with high levels of oxygen and glucose. With the help of these compounds, a lack of other important substrates (purines, cholesterol/steroids, lipids, and amino acids) for the COC might be overcome by increased biosynthesis in cumulus cells. VN, FN, complement C3, A2M and antithrombin III are potential players in equine sperm-oocyte interaction and attraction and were underrepresented after maturation in vitro. These candidates deserve more attention to improve equine IVF success in the future.

Overall, the presented alterations in the “cumulome” after IVM point towards the future direction for the development of more physiological IVM conditions. Fine adjustment of media composition needs to focus on fatty acids, amino acids and purines. This adjustment will contribute to overcome the need for supraphysiological oxygen and glucose concentrations, which seem to aid the COC only by compensating for actual shortcomings in media.

## Methods

### In vivo COC collection

Oestrous was induced by injection of 1 ml Cloprostenol (Estrumate®, MSD Animal Health GmbH, Luzern, Switzerland) in mares (*n* = 7) owned by the University of Zurich that were designated for slaughter for non-reproductive reasons (Additional file 4: Table S4). Mares were regularly checked by transrectal ultrasonography; when a follicle over 35 mm along with uterine oedema was detected, ovulation was induced by injection of 2500 I.U. hCG (Chorulon®, MSD Animal Health GmbH, Luzern, Switzerland). Slaughter was scheduled 30 h after injection, and ovaries were excised from the carcasses to harvest COCs from the dominant follicle by follicular scraping. As expected, all recovered COCs had a nicely expanded CC. Each COC was washed four times in 100 μl phosphate buffered saline solution containing bovine serum albumin (PBS-BSA). In the last step, oocytes were denuded using The Stripper® (Cooper Surgical Fertility &Genomic Solutions, Malov, Denmark), and 3 μl of the CC was collected for analysis. Denuded oocytes were scanned under an inverted microscope for extrusion of polar bodies. All COCs used in the analysis (*n* = 8) were successfully matured, with extrusion of the first polar body. Animal testing authorisation for collection of in vivo matured oocytes was permitted by the Zurich cantonal veterinary office (authorisation number 153/13).

### In vitro COC collection

Oocytes for IVM (*n* = 7) were collected from mares (*n* = 5) slaughtered at local abattoirs. The animals were out of oestrous and slaughtered for non-reproductive reasons (Additional file 4: Table S4). Ovaries were excised from the carcasses, and COCs from non-dominant follicles were recovered by scraping. Only oocytes with compact cumulus oophorus were selected for the IVM process. All cells in close connection to the oocyte were defined as cumulus oophorus. IVM was performed for 30 h in 30 μl droplets of Advanced DMEM/F12 based maturation medium (Advanced DMEM/F-12, Thermo Fisher Scientific, with 2.5 mM Glutamax, 2.2 g/l NaHCO_3_, 1 ml/100 ml foetal bovine serum, 50 ng/ml EGF, 10 IU/ml PMSG and 5 IU/ml hCG). The maturation rate was 62%. After the IVM period, COCs were treated as described for the in vivo matured group with washing and denudation in PBS-BSA. Only cumulus samples from successfully matured oocytes were used for this study. All cumulus samples were snap frozen and stored in liquid nitrogen until preparation for “cumulomics” analysis.

### Untargeted metabolomics analysis

Metabolites of single CCs were profiled using nano high-performance liquid chromatography mass spectrometry (UPLC–nanoESI-MS) in negative mode similar to that suggested by Paglia et al. [142]. In brief, cells were lysed and extracted in 100 μl MeOH/H_2_O (9:1 v:v) using 4 freeze/thaw cycles (1 min liquid nitrogen; 5 min high intensity focused ultrasound (HIFU)). After centrifugation (15 min at 13000 rpm, at 4 °C), the supernatant with lipids and metabolites was collected and stored at − 20 °C. The pellet was collected and immediately processed for proteomics analyses (see below). Prior to analysis, the extracts were dried under a stream of nitrogen and reconstituted in 20 μl water, further diluted with 80 μl 50 mM ammonium acetate in acetonitrile/MeOH (90:9 v:v) adjusted with ammonium hydroxide to pH 9. Metabolites were separated on nanoAcquity UPLC (Waters) equipped with a BEH-Amide capillary column (200 μm × 150 mm, 1.7 μm particle size, Waters) by applying a gradient of 0.5 mM ammonium acetate in water adjusted with ammonium hydroxide to pH 9 (A) and 0.5 mM ammonium acetate in acetonitrile adjusted with ammonium hydroxide to pH 9 (B) from 90% B to 50% B. The injection volume was 1 μl. The UPLC was coupled to a Synapt HDMS G2 mass spectrometer (Waters) by a nanoESI source. MS data were acquired using negative polarization and all ion fragmentation (MS^E^) over a mass range of 50 to 1200 m/z at a resolution of 22,000 (MS and MSMS). All solvents used were of quality HPLC grade (Chromasolv, Sigma-Aldrich). Metabolite data sets were evaluated with Progenesis QI software (Nonlinear Dynamics, A Waters Company), which aligns the ion intensity maps based on a reference data set, followed by peak picking on an aggregated ion intensity map. Detected ions were identified based on accurate mass, and detected adduct patterns and isotope patterns by comparison with entries in the Human Metabolome Database (HMDB). A mass accuracy tolerance of 0.025 Da was set for the searches. Fragmentation patterns were considered for the identifications of metabolites. Quality controls were run on pooled samples and reference compound mixtures to determine technical accuracy and stability.

### Proteomics analysis

After initial methanol extraction for metabolomics analysis, the pellet was immediately prepared for proteomic analysis. Therefore, a sonoreactor-based cell lysis protocol (SR) [143] was combined with FASP (adapted from [39]). The SR-FASP protocol was specifically developed for the analysis of the proteome for single COCs [40]. The pellet was dissolved in 30 μl SDS lysis buffer (4% SDS, 100 mM Tris/HCL pH 8.2, 0.1 M DTT–dithiothreitol) and incubated at 95 °C for 5 min. In the next step, samples were treated with HIFU for 10 min with amplitude of 65% in cycle 0.5 (Sonoreactor UTR200; Hielscher Ultrasonics GmbH). After cell lysis, protein concentration was estimated with a Qubit® Protein Assay Kit (Life Technologies). A total of 10 μg of proteins were used for the adapted FASP protocol [39]. Proteins were diluted in 200 μl UT buffer (Urea 8 M in 100 nM Tris/HCL, pH 8.2) and loaded on a Microcon-30 kDa Centrifugal Filter Unit with Ultracel-30 membrane (Merck Millipore). The unit was centrifuged at 14,000 g for 25 min at room temperature. A wash with 200 μl UT buffer followed by centrifugation at 14,000 g for 25 min was performed. Reduced proteins were alkylated with 100 μl iodoacetamide 0.05 M in UT buffer during an incubation of 5 min, followed by three washing steps with 199 μl UT and two steps with 100 μl NaCl 0.5 M. Protein digestion on the filter unit was performed overnight in a wet chamber at room temperature using 120 μl 0.05 M triethylammonium bicarbonate buffer (pH 8.5) with trypsin (Promega) in a ratio of 1:50 (w/w). After elution at 14,000 g, the peptide solution was acidified using trifluoroacetic acid (TFA) to a final concentration of 0.5%. Peptides were desalted using Finisterre solid phase extraction C18 columns (Teknokroma), dried in a vacuum concentration and resolubilized in LC-MS solution (3% acetonitrile, 0.1% formic acid).

Analysis of all biological samples (*n* = 15) was performed in one analytical run in random order using reverse-phaseLC-MS/MS on an Orbitrap Fusion mass spectrometer (Thermo Scientific) coupled to a nano HPCL system (EASY-nLC 1000, Thermo Scientific) in data dependent acquisition (DDA) mode. A homemade frit-column (75 μm × 150 mm) packed with reverse phase material (ReproSil-Pur 120, C18-AQ, 1.9 μm (Dr. Maisch HPLC GmbH)) was coupled to the MS with a fused-silica spray emitter (20 μm × 8 cm, tip: 10 ± 1 μm; New Objective). A defined amount of 500 ng peptides per sample was loaded to the column and analyzed by LC-MS/MS. For channel A, the solvent composition was 0.1% formic acid in water, and for channel B, it was 0.1% formic acid in acetonitrile. Elution of peptides was performed using a flow rate of 300 nl/min with a gradient of 1 to 35% acetonitrile over 120 min, followed by a cleaning period for 10 min with 98% acetonitrile. Full-scan mass spectra (300–1500 m/z) were acquired with a resolution of 60,000 at 200 m/z after accumulation to a target value of 5e5. Look mass correction (371,1010 and 445,12,003 m/z) was used for internal calibration, and the maximum cycle time between precursor masses was set to 3 s. Data dependent MS/MS were recorded in a linear ion trap using quadrupole isolation in a window of 0.7 Da. Selected ions were fragmented with 30% fragmentation energy. The ion trap was run in rapid scan mode with 1e2 as the target value and a maximum injection time of 35 ms. Precursor ions with charge state from + 2 to + 6 and a signal intensity of at least 1e4 were selected for fragmentation. For 25 s, a dynamic exclusion list was applied with activation of maximum parallelizing ion injections. As reference, a pool containing 5 μl of each cumulus sample was analysed in the same analytical run and used as reference for aligning in data analysis.

Label-free quantification was conducted with Progenesis QI for Proteomics Software (Nonlinear Dynamics, A Waters Company). The reference for automatic aligning was the raw-file of the sample pool. For peak picking, the high sensitivity option was chosen, and only peptides with charge 2, 3 and 4 were used for analysis. The top five mass spectra were exported for a database search using charge deconvolution and deisotoping options with a minimum fragment count of 200 peaks per MS/MS [144]. Spectra were searched against the NCBI data base for horses (NCBI Taxonomy ID 9796, release date 20,170,523). For functional downstream analysis of proteins, the database was blasted to human homologous proteins from the canonical UniProt database (Tax ID: 9606, 20,161,209: file: fgcz_9606_reviewed_cnl_contaminantNoHumanCont_20,161,209.fasta). The database was concatenated with reversed sequence information for estimation of the false discovery rate [144]. The search was run on Mascot Server v.2.4.3. (Matrix Science), with a tolerance of 10 ppm for precursor ion mass and 0.5 Da for fragment ion tolerance. Enzyme specificity was restricted to trypsin with an allowed maximum of 2 missed cleavage sites. As fixed modification, only carbamidomethylation of cysteine was specified, and for variable modifications, deamidation of glutamine and asparagine as well as protein n-terminus acetylation were selected. Protein probabilities by the protein prophet algorithm [145] were analysed in Scaffold v4.1.1 (Proteome Software Inc.). Proteins containing similar peptides that could not be discriminated by the MS/MS analysis were grouped according to the principles of parsimony. To link the MS1 features in Progenesis QI for proteomics with peptide and protein information, we uploaded a Scaffold spectrum report filtered for false discovery rates on the peptide (5%) and protein (10%) levels. The overall false discovery rate for quantifiable proteins with at least two peptides was estimated at 0.2% using the target-decoy strategy [144]. For protein quantification, the average of the normalised abundance from the most intense 3 peptide ions of each protein group were calculated individually for each sample [146]. This generates the normalised quantitative protein abundance. Statistical testing was performed on hyperbolic arcsine transformed values using ANOVA. Differentially expressed proteins were defined with a fold change > 2 along with *p* ≤ 0.05. Enrichment analysis of overexpressed proteins in KEGG pathways was performed online using STRING-Database (http://string-db.org) [147]. All mass spectrometry proteomics data were handled using the local laboratory information management system [148] and all relevant data have been deposited in the ProteomeXchange Consortium via the PRIDE partner repository with the dataset identifier PXD011086 [149].

Integrated analysis of proteomic and metabolomic results was performed using KEGG Mapper v3.1 (release date October 1st 2017; http://www.genome.jp/kegg/mapper.html).

## Additional files

Additional file 1: **Table S1.** All 216 differentially expressed proteins (*p* ≤ 0.05; FC ≥ 2); 204 with unique UniProt IDs. Of these proteins, 95 were significantly underexpressed in the in vitro matured group, and 86 of these proteins were linked to a unique orthologous human UniProt ID (Fig. 1). In the in vitro group, 121 proteins (118 with unique orthologous human UniProt IDs) were significantly overexpressed. (XLSX 140 kb)

Additional file 2: **Table S2.** All 108 metabolic compounds with a significant different (*p* < 0.05; FC > 2) abundance between the two maturation groups. Here, 84 compounds showed a higher abundance in the in vitro group and 24 compounds a lower abundance compared to those in the in vivo matured group. For 28 compounds, putative metabolite IDs were found (6 with lower abundance and 22 with higher abundance after in vitro maturation). (XLSX 84 kb)

Additional file 3: **Table S3.** All 905 measured metabolomic compounds (Progenesis QI Output Measurements). (XLSX 481 kb)

Additional file 4: **Table S4.** Additional information on donor mares and follicles for the in vivo and in vitro matured groups. Mann-Whitney test indicated no significant difference for mare age (*p* = 0.2) and a significant difference for follicle size (*p* = 0.04) between the two maturation groups. (XLSX 10 kb)

## Data Availability

The full metabolomics dataset (Progenesis QI for metabolomics export) is available as supplemental material (Additional file 3: Table S3). All proteomic data are available in PRIDE-Archive (EMBL-EBI) with the dataset identifier PXD011086 (https://www.ebi.ac.uk/pride/archive/).

## Abbreviations

2PG: 2-phospho-d-glyceric acid
A1AT: Alpha-1-antiproteinase 2 precursor (A1AT)
A2AP: Alpha-2-antiplasmin
A2M: Alpha-2-macroglobulin
AARS: Alanine t-RNA ligase
ACACA: Acetyl-CoA carboxylase 1
ACOD: Acyl-CoA desaturase
ACSL: Long-chain-fatty-acid-CoA ligase 3
ADIPO: Adiponectin
ANT3: Antithrombin 3
APOA1 and 2: Apolipoprotein A1 and 2
APOH: Apolipoprotein H
C3: Complement C3
C4: Complement 4 A/B (C4)
C4BP: C4b-binding protein alpha chain
C5: Complement C5
C7: Complement C7
CC: Cumulus complex
CFB: Complement factor B
CFH: Complement factor H X1
CFI: Complement factor I
CHOND4: Chondroitin-4-sulfate
CLUS: Clusterin
COC: Cumulus oocyte complex
CP: Ceruloplasmin
DAG1: Dystroglycan
dCMP: Deoxycytidine monophosphate
DESI: Desorption electrospray ionization mass spectrometry
DTT: Dithiothreitol
EGFR: Epidermal Growth Factor Receptor
ENPP4: Bis(5′-adenosyl)-triphosphatase
F2: Prothrombin
FABP: Fatty acid binding protein
FASN: Fatty acid synthase
FASP: Filter-aided sample preparation
FDR: False discovery rate
FGA: Fibrinogen alpha chain
FGB: Fibrinogen beta chain
FGG: Fibrinogen gamma chain
FMNH2: Reduced flavin mononucleotide (FMNH2)
FN: Fibronectin
Geranyl-PP: Geranyl Pyrophosphate
GFPT1&2: Glutamine-fructose-6-phosphate aminotransferase 1&2
GJA1: Gap junction alpha 1 protein
GLUT1: Solute Carrier Family 2, facilitated glucose transporter member 1
GMPS: Guanine Monophosphate Synthase
Guano: Guanosine
HBA: Hemoglobin A
HBB: Hemoglobin B
hcG: Human chorionic gonadotrophin
HEMO: Hemopexin
HEP2: Heparin Cofactor 2
Hexose-P: Hexose-Monophosphate
HMB: Hydroxymethylbilane
HMDB: Human metabolome database
HSPG2: Basement membrane-specific heparan sulfate proteoglycan core protein
HTRA1: Serine Protease HTRA1
HypoX: Hypoxanthine
I.U.: International units
IC1: Plasma protease C1 inhibitor
IC1: Plasma protease C1 inhibitor (IC1)
ICSI: Intracytoplasmic sperm injection
ID: Identities
IMDH2: Inosine-5′-monophosphate dehydrogenase 2
IPSP: Plasma serine protease inhibitor
ITIH1–4: Inter-alpha-trypsin-inhibitor heavy chain H1–4
IVF: In vitro fertilization
IVM: In vitro maturation
IVP: In vitro production
KEGG: Kyoto encyclopedia of genes and genomes
KNG: Kininogen-1
MAC: Membrane attack complex
Mt-ND5: NADH-ubiquinone oxidoreductase chain 5
MVD: Diphosphomevalonate decarboxylase (MVD);
Oestrone-S: Oestrone sulfate (Estrone-S)
PANT: Pantetheine
PEP: Phosphoenolpyruvate
PFAS: Phosphoribosylformylglycinamidine synthase
PFK-M: ATP dependent 6-phosphofructokinase muscle type
PHPA: Phosphohydroxypyruvic acid (PHPA)
PLG: Plasminogen
POLR2: DNA-directed RNA polymerases I, II, and III subunit RPABC1
PRPS1: Ribose-phosphate pyrophosphokinase
PURH: Bifunctional purine biosynthesis protein
SERC: Phosphoserine aminotransferase
TF: Transferrin
TFA: Trifluoroacetic acid
UMPS: Uridine 5′-monophosphate synthase
VN: Vitronectin
